# Do ban-the-box policies increase the hiring of applicants with criminal records?

**DOI:** 10.1371/journal.pone.0320736

**Published:** 2025-04-16

**Authors:** Deborah M. Weiss, William Dawson, Ronald McKinley, Lee Webster

**Affiliations:** 1 Center on Law, Business, and Economics, Pritzker School of Law, Northwestern University, Chicago, Illinois, United States of America; 2 University of Texas Medical Branch, Galveston, Texas, United States of America; 3 South Central Family Health Centers, Los Angeles; 4 ISO/TC 304 Healthcare organization management, InGenesis, San Antonio, Texas, United States of America; University of Milano–Bicocca: Universita degli Studi di Milano-Bicocca, Italy

## Abstract

Many United States jurisdictions have enacted Ban-the-Box (BTB) laws that are intended to improve the employment prospects of individuals with criminal records. The best-known feature of BTB statutes is a “screening ban:” employers cannot inquire about a criminal record until they have made a conditional offer of employment. Many BTB statutes contain a less well-known “use prohibition:” employers cannot withdraw a conditional offer based on a criminal record unless that record is sufficiently related to fulfillment of potential job duties. In this paper we provide the first evidence of the association of BTB policies with variation in the progression of candidates through hiring phases after the screening process. We use unique applicant-level data obtained from an employer before and after it voluntarily implemented a BTB policy. We find that the enactment of the BTB policy has little or no association with the rate at which individuals with criminal records survive the candidate assessment process and receive conditional employment offers. Indeed, our findings suggest a modest indication of a negative association between the implementation of BTB policies and the hiring of individuals with prior convictions for specific offenses. The observed pattern could be explained if, after losing access to criminal history, employers increase their reliance on hiring criteria that are correlated to criminal history. We also find that the rate at which individuals with a criminal record survive a final background check does not change after the implementation of the joint BTB policies. We find weak evidence that implementation of the two BTB policies is associated with worse outcomes for individuals with records of more serious offenses.

## 1. Introduction

About one-third of all adults in the United States have some type of criminal record [1]. Individuals with criminal records are less likely to obtain and hold jobs than those with otherwise comparable observed human capital [2,3]. The labor market participation of those with records is of great public policy significance, since gainful employment is strongly associated with lower rates of criminal recidivism [4].

Reduced employment rates for individuals with criminal records may stem in part from employer perceptions. Survey evidence indicates that employers regard a criminal record as a negative signal of future job performance [5–7], although existing evidence suggests that these concerns may be exaggerated [8,9]. The availability of criminal background checks has expanded dramatically over time, and employers have made increasing use of these checks. In 2012, about 69% of organizations conducted criminal background checks on all of their job candidates [10]. Recent evidence suggests that 70% to 80% of job applications include a criminal record question, and a large majority of these are asked early in the application process [11,12]. Lower callback rates for applicants with felony records have been found in audit studies in which actors pose as job applicants [13,14] and in correspondence studies in which researchers send real employers the resumes of fictitious applicants [15]. Audit studies have found lower callback rates even for mere arrests without conviction for low-level offenses [16]. One study found that individuals with records have lower employment in states that provide more access to criminal records [17]. However, a study using establishment-level survey data found that the effect of background checks is not uniform across occupation and employers [18].

To improve employment outcomes for individuals with criminal records, many United States jurisdictions have enacted Ban-the-Box (BTB) laws [19] that regulate various stages of the hiring process. For most employers, the first stage of the hiring cycle, the “screening phase,” is an initial screening by recruiters. In the second stage, a candidate who receives a callback undergoes a “candidate assessment process” that includes interviews, tests, and additional resume review by hiring managers and other interested parties. At the end of this stage, successful candidates receive an offer that is contingent on the results of a background check. In the third and final stage, the “background check stage,” the employer requests a criminal background check and decides whether any information it contains warrants rescinding the contingent offer.

The best-known feature of BTB statutes is a “screening ban.” Employment applications often contain a screening question asking applicants to check a box if they have a criminal record. BTB laws prohibit employers from inquiring about an applicant’s criminal record until they have made a conditional offer of employment. This prohibits not only initial screening questions but subsequent inquiries during the candidate process. In effect, screening bans prohibit employers from obtaining information about a criminal record until they have acquired information that could counterbalance the negative signal of that record. BTB proponents suggest that employers are less likely to view criminal records as conclusively disqualifying after meeting applicants in person: “Rejection is harder once a personal relationship has been formed” [20]. In addition to these legislative efforts, policymakers have encouraged employers to adopt BTB policies voluntarily. An Obama administration initiative that included this pledge was signed by over 100 major employers, including American Airlines, Coca-Cola, Koch Industries, Google, Starbucks, and Walmart [21].

Passing through the screening and candidate assessment processes is of little use to an applicant who is disqualified when a background check is finally run. Many BTB statutes regulate the final background check through a “use prohibition:” employers cannot withdraw a conditional offer based on a criminal record unless that record bears a sufficiently strong relation to fulfillment of the potential job duties [19].

BTB policies have a variety of possible effects on the employment prospects of individuals with criminal records. Correspondence and audit studies that show a criminal record penalty imply that screening bans should have a positive effect at the screening stage: individuals with criminal records should receive more callbacks after screening bans are implemented. However, even at the screening stage, these improvements may come at the cost of increased statistical discrimination against members of groups that are disproportionately likely to have criminal records. Statistical discrimination occurs when employers lack perfect information about applicants and make decisions or judgments based on average group characteristics rather than individual qualifications [22,23]. Of particular concern is increased statistical discrimination against people of color, especially males [15,24].

Screening bans might improve consideration of candidates with records but then unravel during two subsequent phases of the hiring cycle, the candidate assessment process and the final background check. Hiring managers may violate the prohibition on criminal record inquiries during interviews, which are harder to monitor than written screening questions. Hiring managers might also engage in statistical discrimination, increasing their reliance on characteristics that are associated with a criminal record. The effect of a screening ban can be further undone when a background check is completed after a conditional offer, since even BTB statutes that contain a use restriction allow employers some latitude to consider criminal records at this time. Hence, the overall effect of BTB policies on hiring may be very different from their effect on the initial callback rates.

Research on the net effect of BTB on employment has been hampered by challenges of finding data that contains both criminal record and labor market history. Two studies using administrative data find that BTB does not improve the employment outcomes of individuals with criminal records. Jackson and Zhao [25] find that earnings of individuals with records decline slightly after implementation of the Massachusetts BTB. Rose [26] finds no impact of the Seattle BTB law on the employment of individuals with criminal records. In contrast, two other studies have found a positive impact. Shoag and Veuger [27] examine the employment of individuals in high crime districts, finding that it increased meaningfully in some sectors after the passage of BTB, especially among the lowest wage workers. Using 1997 National Longitudinal Survey of Youth data, Craigie [28] finds that the passage of BTB produces an increase in the range of 4% to 18% in public sector employment of individuals with criminal records.

These varying results suggest that BTB policies may be effective in some settings but not in others, in turn raising the question of what factors drive these differential effects. An effect of BTB at the screening stage seems likely on both empirical [13,15] and theoretical grounds, since monitoring compliance on job applications is relatively easy. A better understanding of why BTB does not always have the desired result requires closer examination of how BTB policies affect the hiring process after the screening phase. How do BTB policies affect the number of applicants with a record who progress to a final background check? How do they affect the number of applicants with a record who receive a conditional offer but do not receive a final offer based on the results of their background check? Studying outcomes after screening has been limited by the paucity of internal firm data and no direct evidence exists on these issues.

In this paper we make use of a unique internal employer dataset. Our applicant-level data allows us to examine what happens inside an employer after the screening process. The data was obtained from an employer that voluntarily implemented both a screening ban and a use prohibition. The data contain detailed information on any criminal record found during a background check, including the offense type, such as property crime or vehicular offense, and offense level, or seriousness.

We find that the implementation of the two-part BTB policy has little or no association with the rate at which individuals with criminal records survive the candidate assessment process and receive conditional employment offers. Indeed, we find evidence of a small negative association of the policies with lower offer rates for individuals convicted of DUIs. This suggests that most of the positive effect of the screening ban may be undone during the candidate assessment process. We find that the association of BTB with offer rates varies slightly by offense type and offense level. The offense types whose offer rates decline most after the adoption of BTB are not necessarily those that are most disfavored when offense type is known, suggesting that hiring managers are not widely violating the prohibition against inquiries about a criminal record. A more likely explanation is that hiring managers respond to BTB with some type of statistical discrimination, placing greater reliance on hiring criteria that are correlated to criminal history but that happen to be more correlated with some offenses than others. The type of statistical discrimination that occurs in unclear. However, research indicates that the offense types whose offer rates decline after BTB are correlated with certain negative personality traits. This pattern is consistent with greater reliance by hiring managers on these personality characteristics when criminal records become unavailable.

Our data do not contain information on race and we cannot examine the possible role of statistical discrimination based on race, which may be a factor in our results. We see no sign of statistical discrimination against men, who are more likely to have a criminal record.

We also find that the rate at which individuals with a criminal record survive a final background check does not change after the implementation of the joint BTB policies. We find weak evidence that implementation of the two BTB policies is associated with worse outcomes for individuals with records of more serious offenses.

Our results are subject to several qualifications. Our data is from a single employer in a single sector. The employee pool we examine is more educated than is the population as a whole. We cannot speculate as to how well our results will generalize to other populations. However, our evidence suggests that there may be limits to the value of BTB in improving the employment prospects of individuals with criminal records. Although BTB policies may benefit some workers in some sectors, they do not appear to be effective here, indicating that alternative policy tools should be explored as supplements to BTB policies.

## 2. Data

The data was obtained from the University of Texas Medical Branch, a public academic health science center (the “employer”). Preliminary work on the project was conducted with the approval of the University of Texas Medical Branch Internal Review Board, which further determined that the specific data analysis in this paper was not human subject research since the data had been de-identified and no one on the study team had access to identifiers. Because the research was conducted on de-identified existing data, no subject consent was required.

Prior to September 2014, the employer’s job application contained a question that asked if the applicant had a criminal record. At this time, interviewers were not restricted in the questions they could ask about an applicant’s criminal record. If an applicant received a conditional employment offer, a criminal background check was conducted and could be considered in deciding whether to extend a final offer. Recruiters and hiring managers were given discretion about how to use information about a criminal record at all phases of the hiring process.

In September 2014, the employer voluntarily instituted two new policies. First, the employer removed the criminal record question from its job application and prohibited interviewers during the candidate assessment phase from inquiring about an applicant’s criminal record. Second, the employer continued to conduct background checks after conditional offers were extended but it adopted a comprehensive a set of rules regarding the acceptable use of background checks, helping to provide consistency. The rules were implemented uniformly across all employees so that there is no candidate for a control group. The post-employment background check was conducted by the Chief Human Resource Officer and the Chief of Police, who, where appropriate, consulted with the Chief Legal Officer. The Chief Human Resource Officer held both an MBA in Human Resources Management and a Ph.D in Management. The rules restricted the grounds on which a criminal record could be used to rescind an offer to what the policy called “job-related offenses” in which a strong business need could be shown for offer withdrawal, and required individualized assessment of whether these standards were met.

In the employer’s rules, certain criteria for job-relatedness were position-specific. For example, theft convictions carried significant weight for positions involving financial responsibilities or access to valuable property, while a DUI within the past three years disqualified applicants from positions requiring driving. Drug offenses were closely examined, especially for positions in hospital settings where controlled substances were accessible. Misdemeanor drug use was evaluated with some flexibility but convictions with intent to distribute were treated strictly. Some offenses were treated as relevant across all roles. For example, offenses suggesting a lack of integrity were critical concerns. Lying on the application was an absolute bar. Similarly, to protect patient safety, violent or sexual offenses were near-absolute disqualifiers for any position. More generally, the rules directed officials to consider how long ago the offense occurred, with emphasis on offenses within the last seven years. Repeated offenses or a clear pattern of misconduct were seen as more serious than a single isolated incident. Finally, the rules required individualized assessment and aimed to assess the full scope of an individual’s background rather than focusing on any single offense or factor in isolation.

The data are de-identified records taken from background checks, employee records, and applicant data. The background check and employee records data are the principal focus of our analysis. The applicant data contains information about the application process and could be linked to current employee data but not to the background check data. Because of this limit, its primary value was to provide an understanding of the timing of events during the application process, and it was not part of the main analysis dataset.

We match the background check data to employee record data. Background check data is available between July 2012 and December 2018. The records contain some earlier checks, but our analysis begins as of the date for which we have records for all employees. The date restriction was necessitated by records retention policies. Employee record data is available for the entire range of dates for which the background check data is available. These records were provided for individuals who were or had been employed. They included a unique employee ID, date of birth, sex, education level, and hire/separation dates. The unique person identifiers in the background check data are different from those contained in the employee data but both data sets contain enough information to match the background check data to the employee record data.

Our primary source of data is the background check data, which includes both volunteers and job applicants to whom a conditional offer had been extended. The background check data allow us to identify and exclude volunteers. We are not able to exclude applicants to specialized unit positions. This exclusion would have been desirable since these jobs require more stringent criminal record checks. We include full-time, fixed-term, temporary, and part-time employees.

Some individuals had multiple background checks because they applied more than once within the sample window. These repeat applicants may not have received a final offer after their initial application, may not have accepted a final offer, or may have accepted an offer and separated. Each application in the application progression data is treated as a separate observation. Of the unique individuals in our data, 81.5% appear only once, meaning they applied for a single position. The remaining individuals did apply for multiple positions at different points in time, the vast majority only one other time.

The background check data contained first names that were used to impute gender and some observations also contained education data.

Each applicant observation has the characteristics of an individual at the time he or she received a conditional offer for a particular job and includes the entire criminal record, if any, of the individual at that time, possibly containing multiple offenses. If an individual applied for more than one job, we include each application separately in the data set and do not impute offenses contained in later applications back to earlier applications.

We cannot determine which individuals in the background check data eventually received a final offer, and we define hired applicants as those who later appear in the employee record data. We use this as an approximation of those hired, since we expect the gap between offers received and accepted to remain constant absent some intervening factor. We have 22,946 applications for 18,316 unique individuals—12,260 applications were matched to employee records, from which we infer that they began work.

### 2.1. Background check data

The background check data contain criminal record information for each applicant, including detailed information on both convictions and charges that did not result in conviction. The records were obtained by the employer from a private service. Background checks from these providers have been found in one study to contain a significant number of false negatives [29]. The data have unstructured text fields that contain a description of the offense and sometimes the sentence. The data do not contain the date of the offense.

We count only convictions as criminal records. We hand-code the text fields that describe the offense into two new fields, one indicating the offense level, or its degree of severity, and the other the offense type, such as vehicular, drug-related, etc. The background checks also contain a frequently-missing field indicating whether the offense was a misdemeanor or felony. We use it to confirm the accuracy of our coding of offense level but do not principally rely on it. We exclude minor driving offenses, sometimes known as “correctible” since they can be expunged through driving school. Most correctible offenses are corrected and expunged, and thus never appear on a background check. Approximately 1.0% of all applicants had a correctible misdemeanor offense that was reported. The rate for hired applicants was 0.8%.

Table 1 shows the incidence of total non-correctible offenses separately for all applicants and for applicants who were eventually hired. Approximately 6.7% of applicants were individuals who had some criminal record. Among applicants who began work, the rate was 5.4%.

**Table 1:** Individuals with criminal records by offense level and type.

	Number of individuals		Percent of individuals		Percent of individuals with a record	
	Applicants	Hired	Applicants	Hired	Applicants	Hired
Any Non-Correctible Offense	1,547	663	6.7%	5.4%	100.0%	100.0%
**Offense Level (Maximum)**						
Minor misdemeanor	443	190	1.9%	1.5%	28.6%	12.3%
Serious offense	1,104	473	4.8%	3.9%	71.4%	30.6%
Serious misdemeanor	961	425	4.2%	3.5%	62.1%	27.5%
Felony	143	48	0.6%	0.4%	9.2%	3.1%
**Offense Type**						
Vehicle (non-DUI)	460	189	2.0%	1.6%	29.7%	28.5%
DUI	389	191	1.7%	1.6%	25.1%	28.8%
Property	374	139	1.6%	1.1%	24.2%	21.0%
Public admin/order	254	95	1.1%	0.8%	16.4%	14.3%
Drug	192	75	0.8%	0.6%	12.4%	11.3%
High-risk	189	58	0.8%	0.5%	12.2%	8.7%
Misc. Minor Misd.	116	65	0.5%	0.5%	7.5%	9.8%

*Note*: Applicant data is taken from the background check data. Hired applicants are those matched to the employee records data. The unit is person. Offense level percentages are maximum values and sum to 100%. Offense types are not additive. All figures exclude correctible misdemeanor offenses.

The offense level coding distinguishes three offense levels: minor misdemeanors, serious misdemeanors, and felonies. Minor misdemeanors are non-correctible misdemeanors for which fines are the only potential penalty. Serious misdemeanors are those for which jail is a potential penalty. The percent of applicants with an offense at each of the three levels is 1.9%, 4.2%, and 0.6%, in order of increasing severity. The rates of all three offense levels are lower for hired applicants (Table 1).

Table 1 also indicates the percentage of applicants who have at least one offense of the indicated type—percentages therefore do not sum to one. Vehicular offenses are frequent, both those that involved driving under the influence of alcohol (DUI) and those that did not (non-DUI). For both the class of all applicants and the class of hired applicants, the most common offense types are non-DUI vehicular offenses followed by DUIs. Fewer than 1% of all applicants (0.8%) and hired applicants (0.5%) have what we term high-risk offenses, which includes violent offenses, sex offenses, and offenses involving harm to children.

Table 2 presents the education level of the health system’s employees (Panel 1) and disaggregates the incidence of criminal records by employee education level (Panel 2). The education field is frequently missing in the background check data but is generally present in the employee data. The background check data only contained educational information if the employer separately requested a background check to confirm the applicant’s educational history. We are therefore unable to include education as a control but provide an overview of the education in the employee records to give additional context regarding our sample. The employee sample is well-educated relative to the U.S. population overall: 22.1% of the sample has a graduate or professional degree, and additional 25.8% has a Bachelor’s. For only 16.6% is the highest level of educational attainment a high school education or less. The education levels are consistent with a health system that employs many skilled professionals such as doctors, nurses, and social workers. No worker with less than a high school education has a criminal record. Above this educational level, the percentage of employees with a criminal record is negatively associated with educational level.

**Table 2:** Employee criminal records by demographic groups.

Educational level	Percent of Employees			
Panel 1: Employee demographics				
Less Than HS	0.2%			
High School	16.4%			
Some College	14.2%			
Technical Cert	6.1%			
Associate	15.3%			
Bachelor’s	25.8%			
Graduate Ed	22.1%			
Gender				
Female	72.5%			
Male	27.5%			
Panel 2: Criminal record (highest offense level) by education and gender				
Educational Level	Any non-correctible offense	Minor misdemeanor	Serious misdemeanor	Felony
Less Than High School	<0.01%	–	–	–
High School	9.82%	2.55%	5.99%	1.28%
Some College	10.62%	2.80%	7.08%	0.74%
Technical Certification	10.62%	1.37%	7.88%	1.37%
Associate	6.70%	2.05%	4.38%	0.27%
Bachelor’s	5.19%	1.54%	3.41%	0.24%
Graduate Education	3.02%	1.23%	1.61%	0.19%
Gender				
Female	5.74%	1.73%	3.58%	0.43%
Male	9.57%	2.28%	6.45%	0.84%

*Note*: These data are from employees in the employee records data. The unit is person. Individuals are coded as having an offense at the highest level of offense. The category High School includes individuals who completed a GED.

Among high school graduates in our sample, about 9.8% have at least one non-traffic conviction, while about 5.2% of those with a college education have a conviction.

## 3. Results: association of BTB with hiring

### 3.1. Hiring trends

**Fig 1:**
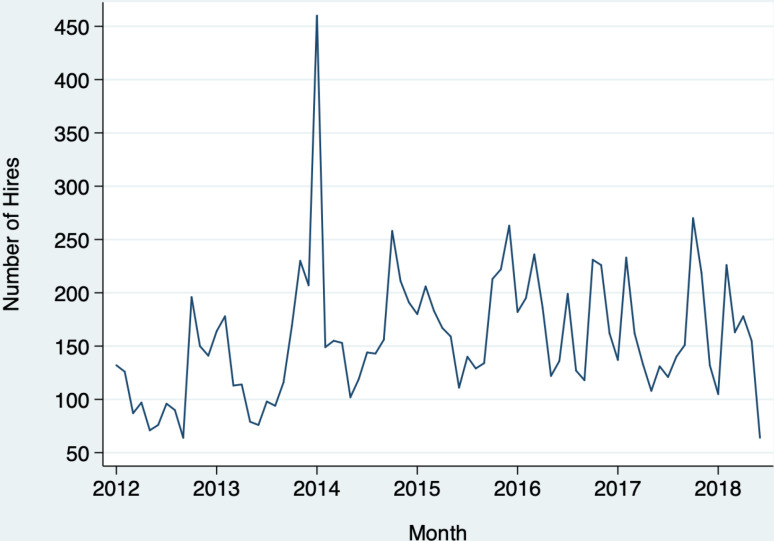
Monthly hires.

In September 2014, the employer instituted a policy of withholding information about criminal records until the time of conditional offer. The month before the new policy went into effect saw a dramatic spike in hiring (Fig 1), suggesting managers anticipated the policy and responded to it with increased hiring. This indicates hiring managers prefer to hire with advance knowledge of applicants’ criminal records.

In 2017, in the middle of the post-policy period, a major natural disaster struck the area. To be cautious we control for the possible effects of the disaster on hiring, but Fig 1 suggests it did not meaningfully affect hiring levels.

### 3.2. Likelihood of offense types and levels at background check before and after BTB adoption

Our background check data consists of applicants who receive a conditional offer of employment triggering a background check. This data includes observations from 25 months before and 52 months after the BTB policy began. Our data contain no information about individual applicants prior to the background report itself. In other words, it contains no information about applicants who applied but did not progress to the conditional offer stage of the application process. We therefore cannot directly examine the effect that, controlling for other factors, having a record would have on individual applicant’s progression to the background check stage. In addition, even for those who received a conditional offer, we do not have general resume-type data, such as experience and education, making it difficult to assess changes in the applicant pool.

Given these limitations, we focus attention on the set of applicants who did receive a conditional offer and examine the association of the policy changes with the likelihood that these applicants had a criminal record. Since we have little individual-level information, we control for certain aggregate factors that might affect the probability of a conditional offer.

We assume that if an employer disfavors applicants with criminal records, the probability that a person with a criminal record receives an offer should decline when the supply of applicants without a record increases. Although we cannot measure the supply of applicants without a record directly, we do know the number of applicants per position. If the proportion of applicants with records remains constant, a rise in total applicants implies a rise in the number of total applicants without a record, giving employers more latitude to reject candidates with records. Our estimates therefore include a three-month rolling average of applicants per position.

Similarly, an employer who disfavors applicants with criminal records but whose demand for labor is increasing may be likely to increase its hiring of individuals with records in order to fill positions. Our measure of labor demand is employer hospital inpatient volume, proxied by total hospital discharges in the employer’s home county, which is commonly used for this purpose since the average hospital stay is about six days [30]. To augment these measures of labor supply and demand, some specifications also use monthly dummies and the one-month lagged unemployment rate for the employer’s home metropolitan statistical area, with no meaningful change in results.

We estimate an equation of the following form on the background check data:


$$\begin{array}{c} Offense=\alpha_{0}+\alpha_{1}Policy+\alpha_{2}Female+\alpha_{3}\left(Female*Policy\right)+\alpha_{4}Age+\alpha_{5}L+\varepsilon \end{array}$$ (1)

where *Offense* is a binary variable taking the value 1 when an applicant has a criminal record of a specified level or type and 0 otherwise; *Policy* is a binary variable taking the value 1 when the applicant’s background check was completed on or after the date of the policy change and 0 otherwise; *Female* is a variable indicating the probability that the applicant is female; Female*Policy is an interaction term; *Age* is the age of the applicant; *L* is a vector of controls including a rolling average of applicants per position, total hospital discharges in the employer’s home county, a binary variable for months affected by the natural disaster, the one-month lagged unemployment rate for the employer’s home metropolitan statistical area, and month dummies; *⍺*_*1*_, *⍺*_*2*,_ *⍺*_*3*,_ *⍺*_*4*_, and *⍺*_*5*_ are parameters to be estimated; and *ε* is a random disturbance term.

We use OLS because the variables of interest are discrete and include interaction terms [31,32]. Note that we do not have data on most of the key variables that might be expected to be associated with a criminal record, including education as well as childhood factors such as home environment. As a result, we do not expect this model to meaningfully predict each applicant’s criminal record—our sole focus is the policy variable.

Table 3 shows the results across levels of criminal records. The dependent variable in each is a dummy variable representing a criminal record of a given offense level. Each estimate includes the observations of individuals with the specified offense level and individuals with no offenses and excludes individuals with other offense levels. The coefficient on *Policy* indicates whether the policy implementation was associated with a change in the probability that an individual applicant who progressed to a background check had the type of criminal record represented by the dependent variable. If the BTB policy works as intended, the coefficient should be significant and positive. The coefficient for any non-correctible offence, representing the net effect of the policy is small, negative, and insignificant. For specific levels of crime, all coefficients are negative, small, and insignificant except in the felony model, where it is borderline significant at the 10% level, indicating that individuals with felony records may be hurt by the policies.

**Table 3:** Likelihood of offense level at background check before and after BTB adoption.

		Type of offense				
	(1)	(2)	(3)	(4)	(5)	(6)
	Any non-correct-ible	Minor misde-meanor	Serious misde-meanor	Serious misde-meanor or felony	Felony	High-risk
Policy dummy	−0.0053	−0.0040	−0.0018	−0.0045	−0.0058+	−0.0005
	(0.0089)	(0.0058)	(0.0075)	(0.0076)	(0.0033)	(0.0035)
Pct female * policy	0.0003	−0.0024	0.0048	0.0055	0.0035	0.0011
	(0.0095)	(0.0063)	(0.0079)	(0.0081)	(0.0036)	(0.0035)
N	20,982	20,982	20,982	20,982	20,982	20,982

Notes: This Table reports OLS regressions, using the background check data, of the probability that an applicant has a particular offense on their record. The reported results include for applicant controls for age and sex and labor market controls for the natural disaster, average applicants per position, average county discharges, and month. The borderline significance of the policy dummy in the felony regression was robust to substituting MSA unemployment for applicants per position as a supply proxy but disappeared when both supply controls were included. Standard errors are clustered at the level of individual applicants whereas the regression itself is run at the level of unique applications—the same individuals do apply for multiple jobs in the data. Clustering standard errors had no discernible impact on the results. The R2 of all estimates is low, ranging from 0.003 to 0.006.

Standard errors in parentheses. + p>0.10 * p>0.05 ** p>0.01 *** p>0.001.

The offer rate reflects the cumulative effect of the screening and candidate assessment phases. If the screening ban is not undone during the candidate assessment process, it should be fully reflected in increased offer rates. Even in the case of felonies, the association we observe is extremely small, 0.58%. Previous work implies that screening bans should increase callback rates by between 4% and 17%. For example, Agan & Starr [33] find that applicants without convictions were 5.2 percentage points more likely to be called back than those with convictions, a 63% increase over the callback rate of 8.2% for those with convictions. Pager [13] finds that white applicants without a criminal record were 17 percentage points more likely to be called back than those without, a 50% increase over the 17% callback rate of whites with criminal records. Any effect might be reversed during subsequent stages of the interview process if hiring managers follow the policy but observe characteristics that are associated with a criminal record or if they violate the policy and inquire about criminal records. Since our data reflects the cumulative effect of the screening and subsequent phases, we assess the power of our estimate using the Stata oneslope command and assume a 2% effect size. For all offense levels the power of our estimate is 99%.

Table 4 shows the association of the policy with changes in the incidence of different offense types. Again, if the policy works as intended, the coefficient on the policy dummy should be positive for all offense types. In fact, the coefficient is positive only for Class C miscellaneous offenses, for which it is significant at only the 10% level and quite small, representing a 0.53% increase in the number of conditional offers observed. The coefficient is negative for all other offense types and insignificant for all but one. For individuals with DUI convictions, it is significant at the 5% level and indicates a 1.15% reduction in the number of DUI records observed at the conditional offer stage (Table 4, column (3)).

**Table 4:** Likelihood of offense type at background check before and after BTB adoption.

		Type of offense				
	(1)	(2)	(3)	(4)	(5)	(6)
	Class C misc.	Drug-related	DUI	Property offenses	Public order/ admin	Vehicular offenses
Policy dummy	0.0053+	−0.0065	−0.0115*	−0.0058	−0.0016	−0.0039
	(0.0029)	(0.0046)	(0.0053)	(0.0044)	(0.0044)	(0.0059)
Pct female * policy	−0.0047+	0.0059	0.0090	0.0028	0.0025	0.0008
	(0.0028)	(0.0047)	(0.0057)	(0.0048)	(0.0045)	(0.0065)
N	19,637	19,705	19,897	19,865	19,757	19,962

Notes: This Table reports OLS regressions, using the background check data, of the probability that an applicant has a particular offense on their record. The reported results include for applicant controls for age and sex and labor market controls for the natural disaster, average applicants per position, average county discharges, and month. The borderline significance of the policy dummy in the felony regression was robust to substituting MSA unemployment for applicants per position as a supply proxy but disappeared when both supply controls were included. Standard errors are clustered at the level of individual applicants whereas the regression itself is run at the level of unique applications—the same individuals do apply for multiple jobs in the data. Clustering standard errors had no discernible impact on the results. The R2 of all estimates is low, ranging from 0.002 to 0.007.

Standard errors in parentheses. + p>0.10 * p>0.05 ** p>0.01 *** p>0.001

Taken as a whole these results do not suggest an aggregate positive association of BTB with changes at the conditional offer stage. For the aggregate “any non-correctible offense” measure, for four out of five offense levels, and for five out of six offense types we find no significant association of the BTB policy with any change in the progression of individuals with a criminal record to a conditional offer. Two coefficients, those for Class C misdemeanors and felonies, are small, borderline significant, and have opposite signs. The only coefficient significant at the 5% level, that for DUI, is negative, the sign opposite to what would be expected if BTB were operating as hoped for.

These results are not necessarily inconsistent with a positive effect of BTB at the screening stage and may indicate that any benefit of BTB at the screening stage is at least partly undone later in the hiring process. Hiring managers who do not have access to information about criminal records might either violate the prohibition on inquiries, engage in statistical discrimination in favor of demographic groups that are less likely to have a criminal record, or search for proxies they believe are associated with a criminal record, such as certain personality traits. We do not have data that allows us to examine statistical discrimination based on race. We do know the sex of the applicant. Female applicants, as shown by the negative coefficient on *Female*, are less likely than males to have a criminal record. If hiring managers responded to the policy by statistically discriminating based on sex, the sign on the interaction between *Female* and *Policy* would be positive, but it is not. As we discuss later, our other results provide some evidence in favor of statistical discrimination by non-demographic proxies and some evidence against hiring manager evasion.

### 3.3. Who is Hired after a Background Check Before and After BTB Adoption?

The employer’s BTB policy included a use restriction that stipulated that a criminal record revealed by a background check could only justify withdrawing a conditional offer if the offense was “job related.” This use restriction is a common feature of BTB laws and policies. Our data reflects the implementation of both a screening ban and a use restriction simultaneously, implying a complex range of possible net outcomes.

Suppose the employer had imposed a use restriction without implementing a screening ban. Continued screening would mean that the employer continued to use a screening question and hiring managers were allowed to ask follow-up questions to be used in the decision to extend a conditional offer. However, the new use restriction would limit the extent to which a hiring manager could use any criminal record revealed in the background check. Because the employer continued to screen, the pool of conditional offerees should contain no more individuals with records than before, and the use restriction should reduce the number of individuals whose offers could be permissibly withdrawn. The effect of the imposition of a use restriction on the progression of individuals with records to employee status should unambiguously be non-negative.

However, our data reflects the implementation of both a screening ban and a use restriction simultaneously. The screening ban might increase the number of individuals with records who progress to a background check, potentially increasing the number of applicants whose offers were withdrawn after the check revealed a record. By shifting the criminal record penalty to the background check phase, the screening ban alone would tend to reduce the number of individuals with records who progressed from background check to final employment.

Thus, the use restriction has a potentially positive effect on policy coefficient while the screening ban has a potentially negative effect. The expected net effect of the joint policy change on progression of individuals with records to employment is therefore ambiguous.

We next examine the association of the joint screening ban and use restriction with a change in the likelihood that those with a criminal record who receive a conditional offer begin employment. We estimate an equation of the following form:


$$\begin{array}{c} Employed=\alpha_{0}+\alpha_{1}Offense+\alpha_{2}Policy+\alpha_{3}\left(Offense*Policy\right)\\ +\alpha_{4}Female+\alpha_{6}Age+\alpha_{7}L+\varepsilon \\ \# \end{array}$$ (2)

where *Employed* is a binary variable taking the value 1 when an applicant began active employment and 0 otherwise; *Offense* is a binary variable taking the value 1 when an applicant has a criminal record of a specified level of severity (any non-correctible offense, serious misdemeanor or felony, violent offense, or high-risk offense) and 0 otherwise; *Policy* is a binary variable taking the value 1 when the applicant’s background check was completed on or after the date of the policy change; Offense*Policy is an interaction term and the corresponding coefficient *α*_*3*_ captures the association of the policy change with outcomes for applicants with criminal records; *Female* is a variable indicating the probability that the applicant is female; *Age* is the age of the applicant; *L* is a vector of controls including a rolling average of applicants per position, total hospital discharges in the employer’s home county, a binary variable for months affected by the natural disaster, the one-month lagged unemployment rate for the employer’s home metropolitan statistical area, month dummies, and dummies for the hiring manager in charge of the individual’s application; *⍺*_*1*_, *⍺*_*2*,_ *⍺*_*3*,_ *⍺*_*4*_, *⍺*_*5*,_ *⍺*_*6*_, and *⍺*_*7*_ are the parameters to be estimated; and *ε* is a random disturbance term. A major reason that individuals who receive a conditional offer do not begin work is non-acceptance of the offer. Since our data do not directly indicate whether a final offer is extended, this model does not predict the conversion of conditional offers to a final offer.

Table 5 shows an estimate of the factors associated with progressing from a conditional offer to starting employment. The policy dummy represents the joint screening ban and use restriction policy. Our sample size permits us to estimate this for a single variable indicating whether the individual had any non-correctible offense. Our data does not have sufficient statistical power to examine how implementation of BTB is associated with the probability that individuals with different levels and types of offenses progress from the conditional offer to ultimate employment.

**Table 5:** Relation between applicant offense and active employment before and after BTB adoption.

	(1)	
Any non-correctible offense	−0.1199	***
	(0.0255)	
Policy dummy	−0.0496	***
	(0.0112)	
Policy*Any non-correctible offense	0.0199	
	(0.0302)	
N	20,982	

*Notes:* This Table reports an OLS regression on applicant data estimating the likelihood that an applicant is hired in the sense of beginning work. The reported results include dummies for the natural disaster, monthly dummies, controls for average applicant per position, and average county discharges. Several controls carry statistically significant estimates, including average applicants per position (which measures relative competitiveness in any given month), county inpatient hospital volume (which measures relative demand for hospital services), and monthly dummies (indicating seasonal variation in hiring patterns). Standard errors are clustered at the level of individual applicants whereas the regression itself is run at the level of unique applications—the same individuals do apply for multiple jobs in the data. Clustering standard errors had no discernible impact on the results. The estimates below are robust to including the metropolitan area unemployment rate. The R^2^ of all estimates ranges from 0.013 to 0.014.

Standard errors in parentheses. + p>0.10; * p>0.05; ** p>0.01; *** p>0.001.

The coefficient on the policy change, shown by interaction of the joint policy change with the non-correctible offense dummy, is not significant, suggesting that the change has no association with the rate at which individuals with criminal records who have progressed to a background check begin employment. In order to assess whether our sample has the statistical power to detect any change, we ask what the lower bound of such an effect might be. If the screening ban is effective and does not simply shift the criminal record penalty to the final phase of hiring, it should increase the hiring of individuals with a record by potentially as much as the criminal record penalty in the range of 4% to 17% found in the earlier audit literature. This might be augmented by a positive effect from the use restriction on criminal records. Assuming this minimum effect size of 4%, our sample has the power at the 0.75 level to detect an association with the joint policy, slightly below the desirable 0.8 level.

## 4. Results: association of offense type and level with the likelihood of beginning employment

Our sample size does not permit us to examine the association of BTB with the probability that individuals with different levels and types of offenses progress from the conditional offer to ultimate employment. However, we can pool the data from the pre- and post-BTB periods and examine how progression to employment varies by offense type and level—a subject of some interest in the previous literature—across our entire sample. These results are helpful in interpreting our findings regarding variation by offense types in the association of BTB with outcomes.

The functional form of the models presented in this section is identical to that presented in Section 3.3 except that the terms relating to the BTB policy are removed. The estimate of the relation between offense type and beginning active employment is shown in Table 6. These estimates do not account for the policy change – that is, they are static across the entire sample period.

**Table 6:** Relation between offense type and active employment.

	(1)	
High-risk offense	−0.1553	***
	(0.0388)	
Class C misc. offenses	0.0601	
	(0.0498)	
Drug offenses	−0.0849	*
	(0.0377)	
DUI offenses	0.0069	
	(0.0275)	
Property offenses	−0.1076	***
	(0.0275)	
Public order/admin offenses	−0.0970	**
	(0.0302)	
Vehicular offenses	−0.0969	***
	(0.0212)	
N	20,982	

*Notes:* This Table reports an OLS regression on applicant data estimating the likelihood that an applicant is hired. The reported results are coefficients on dummy variables for each offense type. The model contains the same set of controls reported in Table 5. R^2^ = 0.014.

Standard errors in parentheses. + p < 0.10; * p < 0.05; ** p < 0.01; *** p < 0.001.

Most offense types are associated with a reduced probability of active employment of about 10%, significant at the 5% level or higher. Individuals with high-risk records are 15.5% less likely to begin employment than those without records, while the association of DUIs with employment is both small and insignificant. The DUI result is interesting when viewed together with the estimated association of the policy with the progression of DUI offenders to the conditional offer stage (Table 4). These results suggest that hiring managers at the employer tend to not penalize applicants for having a DUI on their criminal record. Yet we observe fewer DUI offenders receiving conditional offers after the BTB policy was enacted.

Table 7 shows the association of offense types with the likelihood that an applicant starts work. Column (1) shows the coefficient for all offenses in the aggregate. The point estimate (10.51%) is similar to that presented in Table 5, showing that the inclusion of the policy variables does not meaningfully affect the model estimation.

**Table 7:** Relation between offense level and active employment.

	(1)		(2)		(3)		(4)	
Probability of Active Employment								
Any non-correctible offense	−0.1051	***						
	(0.0137)							
Minor misdemeanor			−0.0916	***	−0.0948	***		
			(0.0211)		(0.0211)			
Serious misdemeanor			−0.0777	***				
			(0.0173)					
Felony			−0.1415	**				
			(0.0432)					
Serious misdemeanor or felony					−0.0902	***		
					(0.0165)			
High-risk offense							−0.1734	***
							(0.0377)	
Non-high-risk offense							−0.0860	***
							(0.0142)	
N	20,982		20,982		20,982		20,982	

Notes: This Table reports an OLS regression on applicant data estimating the likelihood that an applicant is hired. The reported results are coefficients on dummy variables for each offense type. The model contains the same set of controls reported in Table 5. The R2 ranges from 0.012 to 0.013.

Standard errors in parentheses. + p<0.10; * p<0.05; ** p<0.01; *** p<0.001.

Columns (2) through (4) disaggregate the association by offense severity, grouping offense levels in three different ways. Column (2) shows all three severity levels separately. All three have a negative association, significant at least the 5% level. The coefficient on felonies (14.15%) is the highest, although the coefficient on serious misdemeanors (7.77%) is counterintuitively lower than that on minor misdemeanors (9.16%). Column (3) groups serious misdemeanors and felonies together and finds the coefficient on the combined groups to be slightly lower than the coefficient on minor misdemeanors. These departures from expected magnitudes may result from variations in the type of offense predominating in each category.

Column (4) compares high risk offenses with all other offenses. High-risk offenses reduce the probability of actual employment by 17.34% compared to 8.60% for all other offenses grouped together. For the main effects of offense type on employment start there is no prior literature, so our power tests are based on our own observed effect size, which averages to 12%. The statistical power is always over 0.8.

## 5. Discussion and Conclusions

### 5.1. Prior Literature

Improving the employment prospects of individuals with criminal records is an important public policy objective. A growing body of literature has examined whether BTB is a useful tool to achieve that end. Agan and Starr [33] describe the results of a large correspondence study in which real employers received otherwise comparable fictitious applications that were randomly assigned a black or white name and randomly assigned a felony record or none. Applications were submitted before and after the adoption of BTB laws. Prior to BTB, some employers had asked applicants a criminal record screening question. For these employers, Agan and Starr compare the callback rates prior to BTB for candidates with and without a criminal record. They find a 60% higher callback rate for otherwise comparable resumes, implying a corresponding increase in callbacks when BTB laws are passed. In a smaller study, Pager [13] finds a similar effect size of 50%.

Studying what happens after screening is more difficult and has been limited by the paucity of data that contains both criminal record and labor market history. Previous studies that have examined the net effect of BTB on total employment of individuals with records have had mixed results. Two find no effect or negative effects. Jackson and Zhao [25] use Massachusetts administrative data and find that after BTB earnings and employment decline slightly for individuals with pre-existing criminal histories relative to those without. Rose [26] uses administrative employment and conviction data to assess the effect of a Seattle BTB law, concluding that across sectors it had little or no effect on the employment of individuals with criminal records. Rose also finds that in the absence of a BTB law, the negative effect of a criminal record on employment is particularly large in the health sector, but that the effects of BTB were not significantly different across industries.

In contrast, Shoag and Veuger [27] find employment of individuals in high crime districts increased by 3.5% after the passage of BTB. They also find that the effect is concentrated in certain sectors (government, information, education, and real estate) with no statistically discernible effect on the health care sector. This suggests there are meaningful differences across sectors in the impact of BTB. In addition, the effect appears to be greatest among lowest wage workers. Craigie [28] examines data from the 1997 National Longitudinal Survey of Youth and finds a statistically significant increase in the range of 4% to 18% in public sector employment of individuals with criminal records after the introduction of BTB.

At the same time, BTB laws may produce statistical discrimination against groups more likely to have a criminal record. Some studies find that BTB reduces the employment of low-skilled black men [15,24], though the evidence is mixed [27]. Similarly, Doleac and Hansen [24] find that BTB increases the employment of low-skilled Hispanic women, older low-skilled black men, high-skilled black women, and white men, possibly because the lower rate of recent criminal records in these populations creates an incentive for hiring managers to statistically discriminate in their favor. Craigie [28] finds a comparable employment increase for black and white individuals, suggesting low levels of racial discrimination by public employers, perhaps because public employers are subject to stricter discrimination regimes than private employers.

### 5.2. Net Effect of BTB

Our results indicate that for the employer we studied, BTB had little or no association with the progression of individuals with records to conditional offer or to final employment and are in general consistent with the prior literature. Of four previous studies, two find no effect of BTB [25,26] and a third finds effects in many sectors but no effect in the health care sector [27]. In conjunction with prior work, our results suggest that the effects of BTB are uneven across employment settings and are small or nonexistent in some. Since our employer is a public institution, our results are in tension with Craigie [28], but the effect of being a health care provider might dominate that of being a public institution. The apparent sectoral effect may be derivative of effects due to the composition of the workforce. Our employer is a university medical system whose workers are relatively well-educated and well-paid. Evidence that BTB helps lower-income workers [27] suggests that BTB might be more effective in low-paid health occupations such as home health care.

### 5.3. Progression to conditional offer

The novel contribution of our study is the light it sheds on the mechanism by which BTB may sometimes fail to increase employment of individuals with criminal records. The hiring process for this employer, as for many others, can be divided into roughly three parts: screening, candidate assessment, and final background check. The policy change had two parts. The first, the screening ban, eliminated a criminal record question at the screening stage, and prohibited hiring managers or others from inquiring about criminal record. As before the policy change, the background check was only conducted after a conditional offer had been extended and only at this point did the policy allow consideration of a criminal record. We are unable to isolate the role of the screening ban on callback rates, since we only observe the criminal records of applicants once they reach the conditional offer phase and a background check is triggered. However, it seems likely that the BTB policy caused a meaningful increase in applicants with records progressing to candidate assessment. Correspondence studies find an increase in callbacks after BTB of between 50% and 60% [13,15]. In our data, the large jump in hiring before BTB was instituted suggests that recruiters and hiring managers care about criminal records status. Moreover, we find that at the background check stage, both with and without BTB policies, a criminal record is clearly associated with a lower rate of conversion of conditional to final offers. This provides another sign that employers care about criminal records, indicating that BTB might have some effect at the screening stage.

At the same time, we find evidence that any initial effects of BTB are undone for this employer during the candidate assessment phase, since the BTB policy was not followed by a material increase in the number of applicants with criminal records reaching background screening. Across all levels of criminal records, the BTB policy is actually associated with a reduced progression of individuals with records to background screening, although the decrease is significant only for felonies and it is extremely small. For different offense types, the picture is slightly more complex. The intended positive association of the policy with progression is found for Class C miscellaneous offenses, though the effect is small and at a borderline significance level. The coefficient is negative for all other offense types. For individuals with DUI convictions, it is significant at the 5% level and indicates a 1.15% reduction in the number of DUI records observed at the conditional offer stage.

Three factors might undermine the effect of the screening ban during candidate assessment. First, hiring managers might violate the employer’s prohibition on pre-offer inquiries about criminal records [34]. Second, hiring managers might engage in statistical discrimination in favor of demographic groups that are less likely to have a criminal record or search for other proxies associated with a criminal record. Third, hiring managers might increase their reliance on criteria that are correlated with a criminal record.

We have no direct way of observing whether hiring managers violated the prohibition on inquiring about an applicant’s criminal record. However, the negative association of BTB with the progression to a background check of individuals with DUI offenses is suggestive. Our analysis of the association of offense types with progression to employed status indicated that DUIs were the only offense type besides minor misdemeanors to have no association with progression. If managers had been inquiring about criminal records, the pattern observed during the progression to a background check after BTB should more closely resemble the pattern of offenses that resulted in ultimate disqualification, and no negative effect on DUIs should have been observed.

Alternatively, hiring managers might engage in statistical discrimination in favor of demographic groups that are less likely to have a criminal record or search for other proxies associated with a criminal record. Since we observe applicants’ sex but not race, we are able to explore only statistical discrimination based on sex, not on race. We test for gender bias and find no evidence of more women moving to the conditional offer phase after the BTB policy was implemented.

Our observed pattern is consistent with the possibility that after BTB hiring managers increase their reliance on non-demographic factors that are either intended as proxies for criminal history or that are correlated to criminal history. For two offense categories, felonies and DUI convictions, we find that the implementation of BTB actually reduces progression to conditional offer. This change might be explained by the reliance of hiring managers on factors observable to them but not present in our data, such as personality or employment gaps. Research consistently shows that applicant personality affects interview outcomes [35,36]. Research on predicting criminal behavior from psychometrically measured personality traits is focused on relatively specific questions, and broad generalizations are not yet available [37]. However, research suggests that certain personality factors are associated both with a criminal record and with job outcomes [8,9]. DUI and felony offenders frequently display hostile personality traits [38], and if these traits are less common in other offenders, an increased use of personality traits in candidate assessment would produce the observed decrease in DUI offenses or felonies after BTB. Caudy et al. [39] find that substance abusers exhibit different predictors of recidivism than other offenders, again consistent with the hypothesis that hiring managers are relying on personality characteristics that are more common among DUI offenders. Conversely, Class C misdemeanors, which tend to be less serious, might have a lower association with personality traits correlated to criminal behavior, leading to the small positive outcomes associated with the policy.

Our data provides information on a natural experiment in the form of a policy change, allowing us to compare hiring patterns before and after the change. Ideally the experiment would have had an untreated group to which the change did not apply, allowing us to control for secular trends in the hiring patterns. Because our data does not have an untreated group, we address this concern through our controls for various labor supply and demand factors. These still leave room for the possibility of other unobserved factors. For example, it is possible, that the observed decline in DUI offenders with full controls after BTB is the result of fewer DUI offenders overall in the specific metropolitan area where the employer is located. However, the incidence of DUI offenders in our data does not decline year-over-year, as it would with a secular and exogenous decrease in DUIs. We observe a statistically significant decrease in DUI offenses in some, but not all, years after the policy was implemented compared to the years without the policy. Said differently, there is no secular decline in DUI offenses.

A key limitation of our study is that our data is drawn from a single employer. As a result, although we provide evidence that the candidate assessment process *can* undo the initial effects of BTB, we are unable to say how widespread this unraveling is.

### 5.4. Effect of use prohibition

Correspondence studies have been able to isolate the effect of the screening prohibition on callbacks. [13,15]. In contrast, data limitations have presented challenges to examination of the use prohibition, which, once the post-offer background check is obtained, bars employers from considering convictions unless they are “job related.” The use prohibition at the employer in our data went further than this, limiting the power to disqualify based on background checks to the Chief Human Resource Officer, Chief of Police, and Chief Legal Officer. The policy further required these officials to follow guidelines outlining the parameters of job relatedness and business necessity and requiring consideration of individual circumstances. Nonetheless, we find that BTB implementation is not associated with a higher background check survival rate for individuals with records, suggesting that the use prohibition has little effect in our data.

The lack of any association might have several causes. First, hiring managers might have been limiting their screening to job related offenses even before the use prohibition was implemented. Second, even after the policy change, hiring managers might be ignoring both the screening and the use prohibition. In other words, hiring managers might have inquired impermissibly about criminal records and used this information to disqualify based on impermissible factors.

A third and final possible reason for the lack of any observed association is that the use prohibition is at present unclear. The employer in this study implemented detailed guidelines that drew heavily on Equal Employment Opportunity Commission (EEOC) Enforcement Guidance on the meaning of a “job related” conviction in the setting of disparate impact law [40]. It went to great lengths to ensure that three sophisticated and well-trained individuals implemented the guidelines. Despite these precautions, the use restriction has little effect here.

This may signal that restrictions imposed by the use requirement require significantly more elaboration to provide meaningful limits on disqualification. The EEOC provides three examples in which applicants were excluded based on financial offenses from positions involving access to sensitive financial information. In two cases, exclusion is rejected, while in one it is upheld. The explanation accompanying these examples indicates that the EEOC expects employers to rely on research-based evidence and conduct individualized assessments but fails to clarify how these requirements should be balanced or applied consistently. Regarding individualized assessments, it is unclear whether the EEOC believes the burden of producing evidence lies with the employer or the applicant and what threshold must be met for the assessment to satisfy legal standards. The EEOC’s expectations for research-driven policies are similarly vague. In the case where exclusion was upheld, the employer provided recent recidivism data for theft crimes, while the other employers did not offer comparable evidence. The EEOC does not explain why the evidence cited by one employer does not also support the exclusion by the others. Perhaps, though it is not stated, the EEOC expects each employer to independently review or produce social science research. This seems unrealistic and inefficient. Alternatively, the cited research might not apply to the facts of the other examples, yet all involve financial offenses, so some explanation of relevant differences seems necessary. The EEOC does not explain what types of exclusion current research justifies or provide benchmarks for acceptable data. Greater consistency might be achieved if the EEOC regularly updated employers with highly specific guidance on approved research and job-specific exclusions it deems justified.

Taken as a whole, the EEOC’s Enforcement Guidance does not present a clear picture of what is expected of employers. This insufficient guidance may at least in part explain why the implementation of a use prohibition had no effect in our data.

### 5.5. Implications for future research

The existing literature indicates that BTB policies can be effective in some cases but not in others, underscoring the importance of understanding the factors behind these differing outcomes. There is evidence that BTB has an impact at the screening stage [13,15], which is plausible since eliminating screening questions is easy and monitoring employer compliance is relatively straightforward. Prior research has not clarified the extent to which BTB achieves its intended effects beyond the initial screening phase, particularly regarding the number of applicants with a record (i) who advance to a final background check and (ii) whose conditional offer is rescinded due to background check results. Research on these later stages has been constrained by a lack of internal firm data, and this paper is the first to provide direct evidence on these post-screening outcomes.

Potential approaches to studying these questions range from the purely observational to the preferred randomized controlled trial (RCT). A purely observational study would look for correlations between BTB and the two outcomes of interest in a cross-section of companies, some of which had BTB policies and others that did not. Such a study would be limited by the fact that the decision to adopt a BTB policy might be correlated with other employer characteristics. At the other end of the spectrum, an ideal RCT would select a company at random, collect data for an initial period without a BTB policy, impose a BTB policy on the application process of randomly selected job applicants, and study the difference in progression to offers and rescission for individuals with criminal records between the treatment group (those subject to BTB) and the control group (those not subject to BTB).

While an RCT would provide the most rigorous causal evidence, practical and ethical constraints often make it infeasible. A viable alternative is a difference-in-differences (DID) approach, which leverages a naturally occurring policy change—such as adoption of a BTB policy for some applicants but not others—to estimate causal effects. Like the RCT, a DID study could compare progression to offers and rescission before and after a company implements BTB, using applicants not subject to BTB as a control group. Most natural experiments of this kind lack some desirable characteristics of an RCT, such as perfectly random assignment of applicants to treatment and control groups.

However, obtaining internal firm data is notoriously difficult, and achieving the level of collaboration needed for rigorous causal research is even more so. In such circumstances, a strategy of iteration and triangulation is necessary to gradually build toward more precisely identified causal studies.

Because our study examines a policy shock at a single employer, it is somewhat more illuminating than a purely correlational cross-sectional study of companies with and without BTB policies: It compares outcomes at the same company before and after policy adoption, reducing the effects of endogeneity in the decision to adopt a policy that would be present in a purely cross-sectional study. However, the lack of a control group of applications not affected by the policy means we cannot account for changes that might have affected this particular employer during the observed period.

Causal inference exists on a continuum rather than as a binary distinction, and studies that incorporate some elements of causal analysis—such as policy changes—can offer meaningful insights even if they lack all the components required for robust causal identification. Even purely observational studies play a crucial role in hypothesis generation and in refining the focus for further research. Significant effort will be required to find an employer willing to collaborate on an RCT or that has implemented a policy change suitable for DID and is willing to share data. HR systems are complex and imperfect, and even if such employers were identified, obtaining access to both progression and rescission data will be time-consuming. Our study provides evidence that both the progression and rescission stages of the post-screening hiring process warrant the high level of investment needed for a rigorous causal study.

### 5.6. Policy

BTB laws have been widely adopted as a means of facilitating the re-entry of individuals with criminal records into the workforce and may be a valuable tool in some settings. However, our findings suggest that BTB may not be universally effective in achieving this goal. Specifically, our analysis of the two-part structure of BTB – a screening ban at the screening and candidate assessment phases and a use prohibition at the background check phase – suggests that in certain employment contexts the implementation of BTB does not lead to significant changes in outcomes at either of these stages. This finding should be qualified by the voluntary nature of this program, which may have made it less effective than a statutorily-mandated BTB requirement with corresponding enforcement mechanisms

With these qualifications, we believe that our findings point to the value of considering additional policies and interventions at specific phases of the hiring process.

The screening ban operates by allowing applicants with records to prove their value during the candidate assessment phase and works only for individuals who have both the hard and soft skills needed for work [41]. The effectiveness of the screening ban could be improved by investments in hard and soft skills training programs [42,43].

A variety of policies might help individuals with records at the final background check stage. The use prohibition might be improved by a better-developed definition of “job relatedness” that is clearly communicated to employers. Even employer reluctance to hire individuals with job related records might be overcome with financial incentives such as tax credits, bonding, and negligent hiring liability reform [44]. Certain offenses, especially older ones, are unlikely ever to be job related in the sense of predicting problems on the job. In these cases, expanded expungement would take the determination of job relatedness out of employer hands and should reduce the undoing of BTB at the background check stage, an approach supported by evidence finding improved employment prospects after expungement [45]. Evidence of rehabilitation might counteract employer concerns raised by a criminal record, although the evidence is mixed [46,47].

BTB has emerged as an important policy tool in efforts to enhance employment opportunities for individuals with criminal records. Our analysis suggests that BTB may not be sufficient on its own to achieve this goal, particularly in specific sectors and populations, and highlights the importance of a balanced approach that considers a range of policy instruments. To address the complex challenges that individuals with criminal records face in securing employment, we recommend examining specific obstacles at each stage of the hiring process. By identifying and addressing the barriers that impede access of individuals with records to job opportunities, policymakers can develop targeted interventions that complement BTB and draw on the strengths and needs of each group within this population.
